# Understanding of BRCA VUS genetic results by breast cancer specialists

**DOI:** 10.1186/s12885-015-1934-1

**Published:** 2015-11-25

**Authors:** B. K. Eccles, E. Copson, T. Maishman, J. E. Abraham, D. M. Eccles

**Affiliations:** 1 Cancer Sciences Academic Unit and University of Southampton Clinical Trials Unit, Faculty of Medicine, University of Southampton and University Hospital Southampton Foundation Trust, Tremona Road, Southampton, SO16 6YD UK; 2 Cambridge Breast Unit and NIHR Cambridge Biomedical Research Centre, University of Cambridge NHS Foundation Hospitals, Hills Road, Cambridge, UK

**Keywords:** BRCA, VUS, Breast cancer, Specialists, Knowledge

## Abstract

**Background:**

Mainstreaming genetic medicine, increased media coverage and clinical trials for BRCA mutation carriers are leading oncologists into more patient discussions about BRCA genetic testing. *BRCA* variants of uncertain significance (VUS) occur in 10–20 % of tests. VUS detection introduces additional uncertainty for patient and potentially clinician. We aimed to explore the ability of breast cancer specialists (BCS) in the UK to correctly respond to a VUS report.

**Methods:**

A survey sent to 800 UK BCS collected demographics data, VUS general knowledge and interpretation and communication based on two genetics reports. A separate survey of UK clinical geneticists collected demographics data, laboratory reporting practice and methods used to clarify VUS pathogenicity including classification systems.

**Results:**

Of the 155 BCS (22.5 %) who completed the survey, 12 % reported no genetics training. Ninety five percent referred patients for *BRCA* genetic tests, 71 % felt unsure about the clinical implications of the test reports presented here. A VUS report from a patient with a positive family history was interpreted and theoretically communicated correctly by 94 % but when presented with a different VUS report with no management guidance and negative family history, 39 % did not know how to communicate this result to the patient. Geneticists reported multiple VUS classification systems; the most commonly used was word-based in 32 %.

**Conclusions:**

A consistent and standardised format to report particularly VUS results across all diagnostic laboratories plus additional training of UK BCS will be necessary for effective mainstreaming of *BRCA* testing to the oncology clinic.

**Electronic supplementary material:**

The online version of this article (doi:10.1186/s12885-015-1934-1) contains supplementary material, which is available to authorized users.

## Background

Pathogenic mutations in the *BRCA1* and *BRCA2* genes confer a high lifetime risk of breast (and ovarian) cancers [1]. Over the 19 years since the identification of the *BRCA1* and *BRCA2* genes [2, 3]; genetic testing requests to identify pathogenic mutations in *BRCA1* and *BRCA2* have risen steadily. NHS *BRCA* testing in the UK was introduced gradually from the mid 1990’s and has traditionally been delivered through a clinical genetics model driven typically by a strong family history of breast cancer. Demand on the genetics service has increased steadily, with peaks of referral for testing sparked by greater public awareness from press reporting of high profile figures such as Angelina Jolie [4, 5]. A growing interest by oncologists in novel targeted approaches to the management of triple negative breast tumours, together with recognition that this phenotype, (particularly at young ages), is associated with a higher frequency of *BRCA1* mutation carriers, has also increased requests for more rapid access to genetic testing [4]. Young onset triple negative breast cancer cases are eligible for genetic testing even without a family history of breast or ovarian cancer [6]. Faster access to genetic testing without the need for referral to genetics specialists is one of the aims of the “Mainstreaming of genetic testing” agenda [7].

A *BRCA* genetic test can yield 3 possible results: Positive (a pathogenic mutation is found), negative (no mutation detected or a variant of no clinical significance – a polymorphism) or a variant of uncertain significance (VUS). A VUS is an alteration in the gene sequence with unknown consequences on the function of the gene product or risk of causing disease. One classification proposed in 2008 by a workshop of experts convened at the International Agency for Research into Cancer, suggested a 5 point classification scheme following a parallel system to that used in reporting cytology or radiology results [8]. The scheme classifies mutations that are irrefutably pathogenic as class 5, and class 4 variants have a probability of being pathogenic of greater than 95 % taking into account all available data about the variant which may be derived from many different sources. Class 4 and 5 variants can be used for predictive testing in unaffected relatives. A class 3 variant (VUS) has a 0.05–0.949 probability of being pathogenic; evidence may be limited or conflicting and the closer to 95 % probability a variant reaches, the more useful additional family and functional studies are to improve the classification. Therefore Class 3 variants are usually reported out by diagnostic laboratories in order to facilitate further studies. Some of these variants behave as low penetrance gene mutations and should not be managed in the same way as a highly penetrant mutation [9]. This is a complex concept to communicate to a patient who may have undertaken genetic testing with the main aim of gaining access to new treatment options. A class 3 variant would not allow them to be entered into trials aimed at carriers of clearly pathogenic mutations. Most will not have any functional effect but some may have relevance for other family members.

The frequency of VUS reports from different laboratories varies worldwide and depends on testing prevalence and the ancestry of the population served. In African-American populations the rate can be up to 21 %, 5–6 % in individuals of European ancestry in the USA, and 15 % in European laboratories [10, 11]. A record of over 1500 VUS results [12] (as well as pathogenic *BRCA1* and *BRCA2* coding variants) is held in a number of publicly accessible databases. However, these databases vary in how well annotated and curated they are and no public databases currently permit an iterative process of gathering cumulative evidence to reclassify variants. NHS (National Health Service) genetic testing laboratories undergo a national quality assurance scheme to review detection rates of pathogenic mutations and ensure that the Association for Clinical Genetics Science (ACGS) guidelines for assessing the pathogenicity of variants are observed, although these are not specific for the *BRCA* genes [13]. However there is no standard template for *BRCA* reporting and no nationally adopted classification scheme.

Testing recently diagnosed breast cancer patients as opposed to testing unaffected individuals brings unique challenges with complex issues that require careful consideration and a multidisciplinary approach. Common queries include the optimum timing of the genetic test in relation to the patient’s cancer treatment, selecting which patients without a family history require a genetic test and deciding the management of a patient with a very strong family history but a negative *BRCA* genetic test result as well as what to do with a VUS result. In the UK, a 2011 report from the Foundation for Genomics and Population Health (PHG foundation), “Genetics and mainstream medicine” set out a new strategy whereby in the future medical specialities including oncologists will incorporate genetics into their standard practise supported by higher level regional genetics services [7]. The Royal Marsden Hospital is currently piloting the move of *BRCA* genetic testing in breast and ovarian cancer patients away from the genetic clinics into a combined oncology-genetics model. The advantages of more patients having access to genetic tests and a more streamlined process must be balanced with concerns about whether oncologists are prepared or equipped to take on not only the ‘easy’ results but issues such as what to tell the patient when the significance of the result is uncertain.

Previous work has looked at patients’ [14], General Practitioners’ [15, 16] and medical specialists’ knowledge of *BRCA* genetic testing, [17, 18] including oncologists’, [19] but there is little published describing the knowledge of non-geneticist breast cancer specialists particularly with regard to a VUS result. The only study to our knowledge specifically looking at *BRCA* VUS knowledge is a survey of genetic counsellors in the US [20] where significant variation in personal interpretation and management recommendations existed. Another study of patients with a VUS, referring family physicians and genetic counsellors concluded that national VUS-related guidelines were required [21].

In this study we aimed to explore the current knowledge among breast oncologists and breast surgeons at specialist training/registrar and consultant level UK-wide of how to use a VUS result. We explored whether clinicians made correct interpretations and appropriate management choices when presented with two anonymised patient genetics test results taken verbatim from 2 different UK genetics laboratories reporting a VUS result. To place this in context we also approached medical geneticists to gain an insight into the variations in laboratory practice in interpreting and reporting VUS results. This variation in reporting styles from individual laboratories may not be known to individual BCS, but as trainees and consultants work in different UK geographical regions during their career the issue of heterogeneity within UK genetics laboratories is an important point to illustrate.

## Methods

Ethical approval was not required for this survey. Personal, sensitive personal and confidential data was not collected from study participants. Written consent was not required for these quantitative survey, however implied consent was taken by participating in the anonymous survey. Participants were informed that answers were going to be used for publication. The collection method by “survey monkey” and questions meant no identifiable data was collected and the response could not be linked by the researchers to an individual.

### Breast cancer specialists survey - questionnaire 1

A questionnaire was sent electronically in September 2013 to all members of 3 large national organisations: the UK Breast Intergroup, the Association of Cancer Physicians and the Association of Breast Surgeons. Members of these mailing lists included medical and clinical oncologists specialising in breast cancer and breast surgeons, at specialist trainee or consultant level practising within the UK. The breast specialists’ questionnaire was designed by the authors with 10 questions and included questions on demographics, level of genetics training, referral practice for genetic testing, general knowledge of VUS and the interpretation and communication of two anonymised genetic test results reporting a VUS which had been issued by two different UK NHS diagnostic laboratories transcribed verbatim. Additional file 1: Report 1 summary: *“A missense mutation in exon 11 BRCA2 gene (unclassified variant) and change in exon 13 of BRCA2 (rare polymorphism)”*. Additional file 1: Report 2 summary: *“Heterozygous for BRCA2 c.9098C > T, p.Thr3033lle (clinical significance unknown)”*. Full reports and questions are provided in Additional file 1. The questions were close-ended with limitations on multiple responses except for the communication to patients of the two genetics reports. Free text boxes were included to capture further responses and to allow for qualitative analysis.

The term VUS was deliberately not included in the questions about communication of the report to patients, so respondents could not just intelligently guess the answer in the knowledge this was a survey about VUS. The six responses (and other-free text box) were further categorised into appropriate, inappropriate and don’t know responses to allow significance testing between specialities. Appropriate responses, as determined by experts in cancer genetics, were “Explain there may be a hereditary cause and discuss further tests” and “Refer patients to a genetics consultant”. Inappropriate responses were “Reassure the patient that there is no hereditary cause for her breast cancer”, “Explain *BRCA2* mutation contributed to causing her breast cancer”, and “Explain she has a *BRCA2* mutation discuss risk reducing options”. Don’t know also included blank responses. Responses from the free text were reviewed and classified into the appropriate category by BKE.

### Geneticists survey - questionnaire 2

The second survey was also sent electronically from the ENIGMA group (Evidence-based network for the interpretation of germline mutant alleles) to Medical Geneticists in December 2012 and January 2013; only respondents working in the UK have been included.

The geneticists’ questionnaire included study participants demographics questions and level of clinical experience, referral patterns, laboratory workload, *BRCA* and VUS reporting proportions, actions to clarify clinical significance of VUS and classification systems used.

#### Statistical analysis

Analysis was performed in STATA ver. 11.2. Descriptive statistics were used to describe the study population characteristics. Differences between disciplines (oncologist versus surgeon) in Table 2 were tested by Pearson chi squared test. Communication of the report to patients were categorised in dichotomous variables appropriate/inappropriate and ‘don’t know.’ Fishers exact test was used to test differences in communication of report to patients by speciality excluding don't know responses. Free text comments were scored manually and results recorded thematically.

## Results

### Breast cancer specialists survey – questionnaire 1

The breast cancer specialist’s questionnaire was sent to 800 medical and clinical oncologists and breast surgeons of registrar or consultant grade in September 2013 with 181 (22.5 %) responses. Medical oncologists within the UK are specifically trained in the management in cancer using systemic anticancer therapies only. Clinical (radiation) oncologists within the UK are trained in both radiotherapy techniques and systemic anticancer therapies. Responses from allied professionals (nurses/geneticists/radiologists/pathologists/trials staff) were excluded leaving 155 eligible respondents. The most frequent age category was 40–50 years old (34.8 %). Three quarters of respondents were consultants, and three quarters oncologists.

Most specialists (74.2 %) had genetics training in medical school or as part of their postgraduate exams, with 11.6 % stating they had received no genetics training (Table 1). However the majority (95.3 %) had directly referred patients to a genetics service for genetic testing. There was no significant difference between the specialities in terms of genetics training or referral to genetics departments. Overall 71.0 % of respondents felt unsure, uncomfortable or ill-equipped to interpret a genetics report. The distribution of responses was significantly different between surgeons and medical oncologists (*p* = 0.030), but non-significant between surgeons and clinical oncologists (*p* = 0.066) and clinical and medical oncologists (*p* = 0.831). The surgeons were also more confident (50.0 %) than oncologists (24.1 % clinical oncologists and 38.1 % medical oncologists) in perceived understanding of a VUS (surgeons vs medical oncologists *p* = 0.073, surgeons vs clinical oncologist *p* = 0.003) but with no difference in the distribution of responses between oncology specialities (*p* = 0.234). When asked how frequently a VUS is reported all three specialities gave 10–20 % as the most common response.

**Table 1:** Genetics training, referrals and VUS knowledge by breast cancer specialists

	Total	Medical oncologist	Clinical oncologist	Surgeon
	No. (%)	No. (%)	No. (%)	No. (%)
	155 (100 %)	63 (40.7 %)	54 (34.8 %)	38 (24.5 %)
Genetics training				
None	18 (11.6 %)	9 (14.3 %)	5 (9.3 %)	4 (10.5 %)
Medical school/postgrad exams	115 (74.2 %)	47 (74.6 %)	44 (81.5 %)	24 (63.2 %)
Genetics course	4 (3.6 %)	2 (3.2 %)	1 (1.9 %)	1 (2.6 %)
Module with genetics service	1 (0.7 %)	0 (0 %)	1 (1.9 %)	0 (0 %)
Specialist interest (no formal training)	9 (5.8 %)	2 (3.2 %)	1 (1.9 %)	6 (15.8 %)
Higher degree	5 (3.2 %)	2 (3.2 %)	2 (3.7 %)	1 (2.6 %)
Other^a^	3 (1.9 %)	1 (1.6 %)	0 (0 %)	2 (5.3 %)
Direct genetics service referral				
Yes	143 (95.3 %)	59 (93.7 %)	49 (96.1 %)	35 (97.2 %)
No	7 (4.7 %)	4 (6.4 %)	2 (3.9 %)	1 (2.8 %)
Missing	5 (3.2 %)	0 (0 %)	3 (5.3 %)	2 (5.6 %)
Ability to interpret genetics report				
Yes	45 (29.0 %)	14 (22.2 %)	13 (24.1 %)	18 (47.4 %)
No	64 (41.3 %)	27 (42.9 %)	25 (46.3 %)	12 (31.6 %)
Unsure^b^	46 (29.7 %)	22 (34.9 %)	16 (29.6 %)	8 (21.1 %)
VUS understanding				
Fully understand	56 (36.1 %)	24 (38.1 %)	13 (24.1 %)	19 (50.0 %)
Don't fully understand	66 (42.6 %)	25 (39.7 %)	24 (44.4 %)	17 (44.7 %)
Not heard/don't understand	33 (21.3 %)	14 (22.2 %)	17 (31.5 %)	2 (5.3 %)
How common is a VUS?				
<10 %	26 (21.1 %)	9 (17.0 %)	13 (31.0 %)	5 (15.2 %)
10–20 %	61 (47.7 %)	29 (54.7 %)	15 (35.7 %)	17 (51.5 %)
20–30 %	24 (18.8 %)	11 (20.8 %)	8 (19.1 %)	5 (15.2 %)
30–40 %	11 (8.6 %)	4 (7.6 %)	3 (7.1 %)	4 (12.1 %)
40–50 %	2 (1.6 %)	0 (0 %)	2 (4.8 %)	0 (0 %)
>50 %	3 (2.3 %)	0 (0 %)	1 (2.4 %)	2 (6.1 %)
Don't’ know/skipped Q	27 (17.4 %)	10 (15.9 %)	12 (22.2 %)	5 (13.2 %)

^a^“Close cooperation with genetics over last 15 years – personal reading”, “Msc Module” “Experience in Genomics lab”

^b^If 2 answers were given (eg No/Unsure) this was interpreted as ‘Unsure’

Genetics report 1 (a female breast cancer patient with a strong family history) provided the reader with the following summary “missense mutation in exon 11 of the *BRCA2* gene”. In the more detailed interpretation it states that it “has previously been reported as an unclassified variant”, that “this sequence change reduces RAD51C binding activity” and “it may not be appropriate to offer pre-symptomatic testing until pathogenicity has been confirmed” and screening of at risk relatives may help to clarify whether this is a disease-causing mutation in this family”. Only one interpretation of this report was allowed in the questionnaire and the response option most consistent with a VUS was “a gene mutation found but unknown if causing her breast cancer.” A clear majority (83.9 %) chose this response, only 13.6 % responded they “didn’t know” and there were no responses that either a pathogenic *BRCA*2 mutation was found or that no pathogenic mutation was found (Table 2).

**Table 2:** Interpretation of two genetics reports by breast cancer specialists

	Total	Medical oncologist	Clinical oncologist	Surgeon
	No. (%)	No. (%)	No. (%)	No. (%)
	155 (100 %)	63 (40.7 %)	54 (34.8 %)	38 (24.5 %)
Interpretation of report 1				
No pathogenic mutation	0 (0 %)	0 (0 %)	0 (0 %)	0 (0 %)
A gene mutation found but unknown if causing her breast cancer	130 (83.9 %)	57 (90.5 %)	45 (83.3 %)	28 (73.7 %)
A pathogenic BRCA2 gene mutation	0 (0 %)	0 (0 %)	0 (0 %)	0 (0 %)
Don’t know	21 (13.6 %)	5 (7.9 %)	8 (14.8 %)	8 (21.1 %)
Other	4 (2.6 %)	1 (1.6 %)	1 (1.9 %)	2 (5.3 %)
Communication to patient report 1^a^				
Reassure the patient that there is no hereditary cause for her breast cancer.	1 (0.6 %)	0 (0 %)	1 (1.9 %)	0 (0 %)
Explain there may be a hereditary cause and discuss further tests.	95 (61.2 %)	45 (71.4 %)	29 (53.7 %)	21 (55.3 %)
Explain BRCA2 mutation contributed to causing her breast cancer.	0 (0 %)	0 (0 %)	0 (0 %)	0 (0 %)
Explain she has a BRCA2 mutation discuss risk reducing options.	0 (0 %)	0 (0 %)	0 (0 %)	0 (0 %)
Refer patients to a genetics consultant.	81 (52.2 %)	36 (57.1 %)	30 (55.6 %)	15 (39.5 %)
Don't know.	7 (4.5 %)	0 (0 %)	3 (5.6 %)	4 (10.5 %)
Other (free text)	11 (7.1 %)	4 (6.3 %)	2 (3.7 %)	5 (13.2 %)
Interpretation of report 2				
No pathogenic mutation	35 (22.6 %)	18 (28.6 %)	10 (18.5 %)	7 (18.4 %)
A gene mutation found but unknown if causing her breast cancer	71 (45.8 %)	31 (49.2 %)	23 (42.6 %)	17 (44.7 %)
A pathogenic BRCA2 gene mutation	0 (0 %)	0 (0 %)	0 (0 %)	0 (0 %)
Don't Know	49 (31.6 %)	14 (22.2 %)	21 (38.9 %)	14 (36.8 %)
Communication to patient report 2^a^				
Reassure the patient that there is no hereditary cause for her breast cancer.	10 (6.5 %)	2 (3.2 %)	2 (3.7 %)	6 (15.8 %)
Explain there may be a hereditary cause and discuss further tests.	13 (8.4 %)	7 (11.1 %)	3 (5.6 %)	3 (7.9 %)
Explain BRCA2 mutation contributed to causing her breast cancer.	1 (0.6 %)	0 (0 %)	1 (1.9 %)	0 (0 %)
Explain she has a BRCA2 mutation discuss risk reducing options.	0 (0 %)	0 (0 %)	0 (0 %)	0 (0 %)
Refer patients to a genetics consultant.	68 (43.8 %)	24 (38.1 %)	24 (44.4 %)	14 (36.8 %)
Don't know/no answer	58 (37.4 %)	25 (39.7 %)	22 (40.7 %)	11 (28.9 %)
Other (free text)	12 (7.7 %)	5 (7.9 %)	2 (3.7 %)	5 (13.2 %)

^a^Numbers do not add to 100 % as multiple responses allowed

When asked how report 1 would be communicated by the specialist to the patient and future management options any number of responses were allowed (Table 2). Most specialists (61.2 %) would have explained to the patient there may be a hereditary cause and discuss further tests, as consistent with the lab report interpretation provided. Most would refer the patient to a genetics consultant (52.2 %) and only 4.5 % said they “didn’t know”. After categorising the responses of communication to patients for report 1 (Table 3), 94.2 % of specialists gave appropriate responses with statistical significance between speciality responses (surgeon versus medical oncologists *p* = 0.024, surgeon versus clinical oncologists *p* = 0.675, clinical oncologists versus medical oncologists *p* = 0.171).

**Table 3:** Categorising communication to patient responses by breast cancer specialists

	Total	Medical oncologist	Clinical oncologist	Surgeon
	No. (%)	No. (%)	No. (%)	No. (%)
	155 (100 %)	63 (40.7 %)	54 (34.8 %)	38 (24.5 %)
Report 1				
Appropriate	146 (94.2 %)	62 (98.4 %)	50 (92.6 %)	34 (89.5 %)
Inappropriate	2 (1.3 %)	1 (1.6 %)^a^	1 (1.9 %)	0 (0 %)
Don’t know	7 (4.5 %)	0 (0 %)	3 (5.6 %)	4 (10.5 %)
Report 2				
Appropriate	83 (53.5 %)	35 (55.6 %)	28 (51.9 %)	20 (52.6 %)
Inappropriate	11 (7.1 %)	2 (3.2 %)	3 (5.6 %)	6 (15.8 %)
Don’t know	61 (39.4 %)^b^	26 (41.3 %)	23 (42.6 %)	12 (31.6 %)

^a^‘Other: Probable pathogenic mutation so advice is on assumption that this is pathogenic ....’

^b^Includes: Other (*n* = 3): “These questions are hard to answer when we don't deal with interpreting these reports day to day” (*n* = 1); “just read about it before seeing patient” (*n* = 1), “why did I send her for genetic testing?? I wouldn't have done so with no FH!!!” (*n* = 1)

Genetics report 2 (from a female breast cancer patient with negative family history) was summarised as: “Heterozygous for *BRCA2* c.9098C > T”. Further information in the report included statements “not previously reported” and “significance unknown”, with no guidance on management. The BCS interpretation of this report was more mixed with 45.8 % stating “a gene mutation found but unknown if causing her breast cancer”, 22.6 % stating no pathogenic mutation found and 31.6 % don’t know (Table 2).

In terms of communication and possible future management of the patient, 43.8 % of BCS would refer her to a genetics consultant and 37.4 % responded “don’t know or no response given.” 6.5 % would reassure the patient there was no hereditary cause for her breast cancer and 8.4 % would say there was a hereditary cause and discuss further tests (Table 2).

Again after all the communication responses were categorised, only 53.5 % were appropriate responses and 39.4 % don't know (Table 3). No significant differences between response by specialty were found (surgeon versus medical oncologists, *p* = 0.084, surgeon versus clinical oncologists *p* = 0.222, and clinical oncologists versus medical oncologists *p* = 0.810). 7.1 % of specialists communicated an inappropriate interpretation to patients. 6.5 % were overly reassuring and 0.6 % would have said a *BRCA2* mutation contributed to causing the breast cancer.

There was more uncertainty around both the interpretation, and communication and management of genetics report 2 in comparison to report 1. Most free text responses for reports 1 and 2 were themed around whether a geneticist would/should be interpreting these results rather than breast cancer specialist as this was a ‘complicated result’. One specialist discussed the pre-test risk being high in report 1 and therefore considering surveillance and risk reducing options despite no clear pathogenic mutation. For report 2 one surgeon said he/she would not have referred this patient without a family history.

### Geneticists survey – questionnaire 2

The geneticist’s survey was sent to the members of the ENIGMA consortium [22] who further distributed the survey and 175 responses were received from 21 countries. Of the 61 medical geneticists who responded we included only UK practising doctors leaving 31 eligible respondents (Table 4). Most (51.6 %) geneticists surveyed were aged 40–49 and had been working in genetics for >10 years and discuss test results directly with patients on one or more occasion per week. Two thirds showed a copy of the test report to the patient. Geneticists reported using multiple methods to clarify VUS significance (median 5, range 0–11). They mainly worked with busy NHS diagnostic genetic laboratories with a 4–8 week time from blood draw to report. Pathogenic mutations were reported more commonly (10–20 %) than VUS (1–10 %) in these UK laboratories. A specific classification system was used in 11/31 (35.5 %) of laboratory reports (for internal use) and 12/31 (38.7 %) of clinical reports (report received by the requesting clinician). The same respondents reported that multiple systems of classification were used by their diagnostic laboratories; the most common was a word based system (pathogenic, uncertain, polymorphic) in both laboratory and clinical reports (32 %), next most frequently used was a 4 point system based on a review of in-silicon and literature evidence (in 13 % laboratory and 3 % clinical reports) and then the 5 point IARC system based on the multifactorial likelihood of pathogenicity, (10 % laboratory and 6 % clinical reports). *BRCA* test requests were accepted from clinicians in a genetics clinic or from allied professions with genetics qualifications. Very few were accepted from clinicians in oncology clinics or from patients.

**Table 4:** Medical geneticists questionnaire results (*n* = 31)

	Number	Percentage
Age category		
30–39	5	16.1 %
40–49	16	51.6 %
50–59	8	25.8 %
60+	2	6.5 %
Length of time working in genetics		
< 5 years	1	3.1 %
5–10 years	8	25.8 %
>10 years	22	71.0 %
Patient contact		
Do you discuss test results directly with patients?		
Never	2	6.5 %
Rarely (1–2/yr)	0	0 %
Sometimes (1–2/month)	4	12.9 %
Regularly (≥1 per week)	25	80.6 %
Do patients see a copy of the BRCA testing report?		
Yes	8	25.8 %
Sometimes	11	35.5 %
No	3	9.7 %
Not sure/missing	9	29.0 %
Acceptance of BRCA test requests^a^	Yes	No
Clinicians in genetics clinic	26 (83.9 %)	0 (0 %)
Clinicians in oncology clinic	3 (9.7 %)	19 (61.3 %)
Primary care/family doctor	1 (3.2 %)	23 (74.2 %)
Allied professionals with genetics qualification	23 (74.2 %)	2 (6.5 %)
Patients	1 (3.2 %)	22 (71.0 %)
Lab capacity and reporting	Most common response	No. (%)
No. of patient samples tested per year	100–500	17 (54.8 %)
Proportion reporting clearly pathogenic mutation	10–20 %	17 (54.8 %)
Proportion reporting a VUS	1–10 %	12 (38.7 %)
Length of test time (blood draw- report)	4–8 weeks	13 (41.0 %)
Methods to clarify significance of a VUS		
Colleague discussion	23	74.2 %
Information from other lab/clinical expert	16	51.6 %
Co-segregation (additional blood from family)	23	74.2 %
Literature search	20	64.5 %
Mutation database search	15	48.4 %
Google search	9	29.0 %
Splicing prediction software	7	22.6 %
Conservation database	7	22.6 %
Tumour pathology report	7	22.6 %
Tumour DNA	5	16.1 %
Other: RNA studies	2	6.5 %

^a^Numbers exceed 100 % as multiple responses allowed

## Discussion

This survey was designed to gain a snapshot of the broad understanding of VUS’s amongst non-geneticist breast cancer specialists in the UK. We tested their interpretation and anticipated patient communication using two actual reports where a VUS had been identified. Our study demonstrates that most breast cancer specialists currently directly refer their patients to genetics service for a *BRCA* test. Perceived genetics knowledge is not uniform over differing specialities although genetics training is mainly limited to undergraduate level or as part of preparation for postgraduate exams. Overall the majority of survey respondents felt uncertain about their ability to understand the implications of the reported variants based on the genetics reports they were presented with. There was a significant difference between specialities with surgeons feeling more confident than medical oncologists (*p* = 0.030). The results from report 2 highlight the potential for wide variation in interpretation of genetics reports by non-geneticists and the uncertainty around management generated particularly in a patient without a family history of breast or ovarian cancer. However this group of patients with for example young onset triple negative breast cancer but no family history are those who are most likely to be tested directly in the oncology clinic as part of the mainstreaming agenda.

The geneticists that responded to the survey reported that their laboratories reported fewer VUS as a proportion of all reports (1–10 %) than might be anticipated from the literature or was thought to be likely by the breast cancer specialists surveyed (10–20 %). This may be because with increasing volumes of tested and reported *BRCA* tests over many years, a previously classified VUS may be more often recognised as polymorphisms in the tested population and therefore no longer reported as a VUS.

We deliberately chose two recent genetic test reports from laboratories where differing presentation styles reflect the variation in UK Genetics laboratories report format. There is no standardised national reporting template and not all laboratories give clear clinical guidance on management (i.e. for a VUS this would be a clear statement that the VUS should NOT be used for predictive testing and that the family history should guide management) [8, 23]. Equally there is no consensus surrounding a single classification method for VUSs within the genetics community [24, 25] as further demonstrated by the results of our geneticists survey where multiple classification systems were used with no clear majority leader. This can lead to further confusion when non-geneticists receive reports potentially from different laboratories. The American College of Medical Genetics and Genomics and the Association for Molecular Pathology have issued a joint guideline for interpretation of sequence variants [26].

Multiple approaches aimed at understanding the pathogenicity of a variant in any gene exist and these are set out in the ACGS guidelines [13]. Some methods involve in-silico analysis but some require additional samples from other affected family members or tumour samples to fully assess [12, 25, 27]. From this survey it can be seen that UK geneticists commonly adhere to guidelines and use multiple methods (median 5). These independent lines of evidence are more powerful if combined and can be used to give a posterior probability of pathogenicity of a *BRCA* VUS with resulting classification that can be linked to clinical actions [20, 25]. Thus, for a VUS, referral onwards to the genetics services is a necessary action; however, it is important for referring clinicians to convey to their patients that most VUS are not pathogenic, most remain of unclear significance and very few end up with sufficient additional evidence to formally move into IARC Class 4 (i.e. sufficiently certain to be used as a predictor of future risk).

Other studies have found varying knowledge of breast cancer genetics by physicians. A survey looking at US oncologist’s general *BRCA* knowledge found only 40 % correctly answered all 4 questions [19]. In contrast a Dutch survey reported better knowledge on hereditary breast cancer by surgeons, medical and radiation oncologists and radiologists with average score of 6.1/7 correctly answered questions [17]. However neither study specifically looked at *BRCA* VUS knowledge. Certainly there is considerable scope for physician (and patient) misunderstanding of *BRCA* results leading to inappropriate patient care [28, 29] and uncertainty about clinical management [23]. In our study most breast cancer specialists correctly interpreted report 1 but found report 2 more challenging with a greater degree of uncertainty.

The additional burden of an uncertain genetic test result during the emotionally charged time of a cancer diagnosis may encourage patients towards risk reducing surgery that may be inappropriate. Several studies report risk reducing surgery rates amongst patients receiving a VUS result. These vary widely from similar to those tested and receiving a negative report 7–10 % RRM [14, 30] to high rates (42 %) within one year after receiving a VUS result [31]. Genetic testing at the time of cancer diagnosis, sometimes called “treatment focused genetic testing” is already part of the mainstreaming agenda for more personalised treatment and is being implemented routinely in some areas of the UK particularly with a view to facilitating entry into clinical trials. The GTEOC study [32] is looking at upfront genetic testing at time of ovarian cancer diagnosis irrespective of family history and the OlympiAD trial [33] is recruiting *BRCA* gene carriers with distant metastases. Trials of targeted therapies in *BRCA* mutation carriers in the adjuvant setting are now recruiting and require early identification of gene carriers.

Patients find that genetic counselling is helpful (>90 %) and reduces cancer distress [14]. Yet genetic counselling services in the NHS have insufficient resource to manage the inevitable increase on demand for rapid testing. Mainstreaming is inevitable and this study demonstrates that there is an urgent need to ensure not only adequate education in germline genetic testing for cancer specialists but also integration of the cancer geneticist into the multidisciplinary team. Agreement of a national reporting template to provide unambiguous test reports to the receiving non-expert clinician with clear instructions for the clinical utilisation of the test result that can be audited is needed.

### Limitations

As with all studies using survey methodology, data collection is limited by selection bias of respondents most interested in the subject matter. It is recognised that the answers in the interpretation of the reports were not mutually exclusive and therefore there may have been more than one “correct” answer depending on the participant’s interpretation.

## Conclusion

We conclude that at present UK non-genetics trained breast cancer specialists would require additional training to competently deliver VUS results directly to the patient and incorporate results into an appropriate management plan. A multi-disciplinary approach will remain crucial. The adoption by all genetics laboratories of a standard reporting template and a single classification system linked to clear clinical actions would greatly assist the safe integration of germline genetic testing into the mainstream of oncology practice.

## Additional file

Additional file 1: **Interpretation and communication to patient of** ***BRCA*** **test report.** **Report 1.** You sent your female breast cancer patient who has a strong family history (mother and aunt) of breast cancer for genetic testing. She returns to clinic and you have to explain this report to her. **Report 2.** You sent your female breast cancer patient who has NO family history of breast cancer for genetic testing. She returns to clinic and you have to explain this report to her. (DOC 26 kb)

## Abbreviations

ACGS: Association for Clinical Genetics Science
BCS: Breast cancer specialists
ENIGMA group: Evidence-based network for the interpretation of germline mutant alleles
NHS: National Health Service
VUS: Variants of uncertain significance
