# Supplementary material for: Understanding of BRCA VUS genetic results by breast cancer specialists
Source: BMC Cancer. 2015 Nov 25;15:936. doi: 10.1186/s12885-015-1934-1 (PMC4660681; doi:10.1186/s12885-015-1934-1)
Supplement: Additional file 1: — Interpretation and communication to patient of BRCA test report. Report 1. You sent your female breast cancer patient who has a strong family history (mother and aunt) of breast cancer for genetic testing. She returns to clinic and you have to explain this report to her. Report 2. You sent your female breast cancer patient who has NO family history of breast cancer for genetic testing. She returns to clinic and you have to explain this report to her. (DOC 26 kb) [file 12885_2015_1934_MOESM1_ESM.docx]

**REPORT 1**

**Q: You sent your female breast cancer patient who has a strong family history (mother and aunt) of breast cancer for genetic testing. She returns to clinic and you have to explain this report to her.**

“Missense mutation (c.4813G>A; p.Gly1529Arg) identified in exon 11 of the BRCA2 gene.

Report interpretation: Sequencing analysis of exon 11 of the BRCA2 mutation gene identified a G to A base substitution at nucleotide position 4813 (c.4813G>A) resulting in the substitution of the amino acid glycine for an arginine at codon 1529 (p.Gly1529Arg). This sequence change has previously been reported as an unclassified variant on the BIC database. A report by Chen et al. (J. Biol.Chem., 274, 32931) has shown that this sequence change reduces RAD51 binding activity. It may not be appropriate to offer presymptomatic testing to at-risk relatives until pathogenicity has been confirmed. Screening of affected relatives may help to clarify whether this is a disease-causing mutation in this family. No other mutation was identified in with BRCA 1 or BRCA2. Please note it has only been possible to screen fragment 11.5 of BRCA2 in one direction. For information a synonymous change has been identified in exon 13 of BRCA2 (c.7206T>G, p.SEr2326Ser). This change has not been reported in the literature of on the ENSEMBL or Genbank databases, but is likely to be a rare polymorphism.”

What do you think this report means? (tick one only)

- No pathogenic gene mutation found.
- A gene mutation found but unknown if causing her breast cancer.
- A pathogenic BRCA2 gene mutation found that is causing her breast cancer.
- Don’t know /unsure exactly what this result means.

What are you going to say to her? (tick as many as apply)

- Reassure the patient that there is no hereditary cause for her breast cancer.
- Explain there may be a hereditary cause and discuss further tests (eg screening cancer affected relatives).
- Explain she has a BRCA2 mutation that has contributed to causing her breast cancer.
- Explain she has a BRCA 2 mutation and now / in the future discuss risk reducing options.
- Refer patient to a genetics consultant.
- Don’t know.

**REPORT 2**

**Q: You sent your female breast cancer patient who has NO family history of breast cancer for genetic testing. She returns to clinic and you have to explain this report to her.**

“BRCA1 and BRCA2 dosage analysis: BRCA1 and BRCA2 gene analysis:

Normal Heterozygous for BRCA2 c.9098C>T, p.Thr3033Ile

Patient X has been screened for mutations in all exons of the BRCA1 and BRCA2 genes. A C>T substitution at nucleotide 9098, c.9098C>T, was detected in exon 23 of the BRCA2 gene. This substitution changes codon 3033 from a threonine residue to a isoleucine, p.Thr3033Ile. This amino acid changes has not been previously reported on the BIC or HGMD databases therefore the clinical significance of it is unknown. The splice site predictor program (www.fruitfly.org/cgi-bin/seq_tools/splice.pl) did not predict any donor or acceptor site alteration. No other mutation was detected and there was no evidence of a whole exon deletion or duplication of the BRCA1 or BRCA2 gene using MLPA dosage analysis.”

What do you think this report means? (tick one only)

- No pathogenic gene mutation found.
- A gene mutation found but unknown if causing her breast cancer.
- A pathogenic BRCA2 gene mutation found that is causing her breast cancer.
- Don’t know /unsure exactly what this result means.

What are you going to say to her? (tick as many as apply)

- Reassure the patient that there is no hereditary cause for her breast cancer.
- Explain there may be a hereditary cause and discuss further tests (eg screening cancer affected relatives).
- Explain she has a BRCA2 mutation that has contributed to causing her breast cancer. Explain she has a BRCA 2 mutation and now or in the future discuss risk reducing options (eg mastectomy).
- Refer patient to a genetics consultant.
- Don’t know.
